# The contribution of community health systems to resilience: Case study of the response to the drought in Ethiopia

**DOI:** 10.7189/jogh.12.14001

**Published:** 2022-10-23

**Authors:** Angeli Rawat, Jonas Karlstrom, Agazi Ameha, Macoura Oulare, Mohamed Diaaeldin Omer, Hentsa Haddush Desta, Shalini Bahuguna, Katrina Hsu, Nathan P Miller, Gemu Tiru Bati, Kumanan Rasanathan

**Affiliations:** 1 School of Population and Public Health, University of British Columbia, Vancouver, Canada; 2 UNICEF, New York, New York, USA; 3 UNICEF Ethiopia Country Office, Addis Ababa, Ethiopia; 4 Federal Ministry of Health of Ethiopia, Addis Ababa, Ethiopia; 5 Providence Health Care, Vancouver, Canada

## Abstract

**Background:**

Ethiopia’s exposure to the El Niño drought (2015-2016) resulted in high malnutrition, internally displaced people, and epidemics of communicable diseases, all of which strained the health system. The drought was especially challenging for mothers and children. We aimed to identify salient factors that can improve health system resilience by exploring the successes and challenges experienced by a community-based health system during the drought response.

**Methods:**

We collected data via key informant interviews and focus group discussions to capture diverse perspectives across the health system (eg, international, national, district, facility, and community perspectives). Data were collected from communities in drought-affected regions of: 1) Somali, Sitti Zone, 2) Hawassa, Southern Nations, Nationalities, and Peoples’ Region (SNNPR), and 3) Tigray, Eastern Zone. Data were analysed using a deductive-inductive approach using thematic content analysis applied to a conceptual framework.

**Results:**

A total of 94 participants were included (71 from the communities and 23 from other levels). Key themes included the importance of: 1) organized community groups linked to the health system, 2) an effective community health workforce within strong health systems, 3) adaptable human resource structures and service delivery models, 4) training and preparedness, and 5) strong government leadership with decentralized decision making.

**Conclusions:**

The results of this study provide insights from across the health system into the successes and challenges of building resilience in community-based health systems in Ethiopia during the drought. As climate change exacerbates extreme weather events, further research is needed to understand the determinants of building resilience from a variety of shocks in multiple contexts, especially focusing on harnessing the power of communities as reservoirs of resilience.

Climate change has resulted in extreme weather events that have adverse outcomes on global population health; these patterns are expected to continue, especially in areas where resources are relatively scarce [1]. One such extreme weather event is droughts, which create health-related vulnerabilities in populations, and can exacerbate rates of communicable disease, protracted displacement, mental illness, and chronic illness [2]. This can place additional strains on health systems, especially for service delivery for maternal, newborn, and child health (MNCH).

Ethiopia has experienced increasingly severe weather conditions over the past 30 years, the most severe of which was the El Niño drought of 2015-2016. The drought resulted in an estimated loss of 80% of the harvest (2015) and eight million people in need of food assistance (400 000 children at risk of severe malnutrition; 700 000 breastfeeding and pregnant women, and one million children at risk for moderate malnutrition) (2016) [3]. Drought-affected communities experienced increased under-5 death rates, wasting and diarrheal incidence, and anaemia rates of nearly 30% in children [4-6].

Although Ethiopia has reduced child and maternal health morbidities, challenges remain. These challenges include a lack of access to equitable, high-quality health service delivery and ongoing issues of poverty, malnutrition, low parental education, and high fertility rates, all of which contribute to poorer maternal and child health outcomes [7]. The health system has historically been characterized by deficits in human resources, poor access to basic health services, and low availability of health facilities, especially in rural areas, which are home to over 85% of the population [7]. To address this gap and offer priority health interventions for MNCH, the Ethiopian government reoriented the country’s health system towards community-based primary health care in 2003/2004.

In 2003, the Ethiopian government implemented the Health Extension Program (HEP), which deployed Health Extension Workers (HEWs) to strengthen community-based health systems in rural populations [8]. HEWs are paid government employees based partly in communities and in health posts, with diverse roles and responsibilities in some regions. They are supported in their position by community groups such as the health development army volunteers (HDAVs), which are comprised of community women who have demonstrated exemplary health behaviours.

The HEW program has demonstrated mixed results in improving MNCH care in Ethiopia. While visits from HEWs positively influenced antenatal care (ANC) utilization, there has been little or no improvement in facility birth rates or coverage of essential newborn care, despite increases in deliveries attended by skilled birth attendants [9-12]. Some studies in the postnatal period have indicated that health care utilization increased with the presence of HEW programs, while others found no impact [9,10,13]. Similarly, no impact was observed for integrated community case management (iCCM) of childhood illness, despite the high quality of care provided by HEWs [14].

Limited health service utilization and low numbers of household visits by HEWs have been reported as barriers to the success of the HEW program [13,15]; ongoing low immunization coverage [16] and inequities in MNCH service delivery [17] make it critical for understanding how HEWs and the communities they serve can best be leveraged to build resilience in health systems, especially in times of shock.

Building resilience in health systems has garnered increasing attention considering new and emerging threats or shocks to health systems globally, many of which have occurred simultaneously or in succession (eg, Ebola Virus and COVID-19, natural disasters, economic and security crises). However, the lack of clarity in the conceptualization of resilience (and how to operationalize it) remains a challenge [18]. Resilience has been defined by Kruk et al. [19] as the “capacity of health actors, institutions, and populations to prepare for and effectively respond to crises; maintain core functions when a crisis hits; and, informed by lessons learned during the crisis, reorganize if conditions require it”. Some describe resilience as an emergent property which frames health-related challenges within a systemic approach that recognizes the dynamic and interconnected nature of health systems [20-22]. Others warn that the resilience paradigm promotes a form of neoliberal governmentality which places the responsibility to “bounce back” from crises on individuals and communities while ignoring the political forces shaping the crisis [23,24]. Advocating a health system “bounce back” to states of structural weakness and social inequalities may hide a potentially deficient status quo [25,26]. The drought response in Ethiopia provides a unique opportunity to add to the evidence base for health system resilience by examining the successes and challenges experienced across the health systems in the country. Such evidence could inform efforts to strengthen health systems and help build resilience through maximizing resources and support for improved maternal and child health, both in times of crisis and beyond.

We aimed to identify salient factors that can improve health system resilience by exploring the successes and challenges experienced by a community-based health system during drought response. Sub-objectives included understanding barriers and facilitators to community engagement and participation in the health system at the community level and barriers and facilitators to HEWs’ effectiveness, including how their role changed from before the drought to during the drought response, especially in the provision of MNCH services.

## METHODS

Focus group discussions (FGDs) and key informant interviews (KIIs) were conducted to meet the objectives. FGDs were employed primarily at the community level while KIIs were conducted mostly at the district, sub-national, or national levels. This study was part of a four-country study on building resilience in community-based health systems.

### Data collection, participant recruitment and selection

The UNICEF country office, in partnership with the Federal Ministry of Health (FMOH), identified three diverse drought-impacted geographic areas for the community-level data collection: 1) Sitti Zone, Somali Region, 2) Hawasa Zone, Southern Nations, Nationalities, and Peoples’ Region (SNNPR), and 3) Eastern Zone, Tigray Region. These zones were selected to gain diverse insights on health system-related challenges in the various regions of Ethiopia. We purposively selected areas that had recently experienced drought to include agrarian and developing regions (those with limited infrastructure) and urban, peri-urban, and rural areas.

Purposive sampling was used to identify the first round of participants. Participants were then recruited via snowball sampling by UNICEF staff members with the goal of capturing diversity across the health system (ie, from lowest to highest level and key players within these levels) and geographically. Community-level participants included HEWs, village and community leaders, women’s HDAVs, primary health care unit (PHCU) health workers, and mobile health and nutrition unit staff. Non-community participants included district health staff and management, FMOH representatives, multilateral health organizations, bilateral development partners, and international non-governmental organizations (NGOs). Participants were approached in person or by phone and were read a script, informed about the goals and research team, and given the opportunity to ask questions. All participants provided written informed consent. In cases where participants were illiterate or had low literacy, consent forms were read to them, and thumbprints were collected in lieu of signatures.

The conceptual framework guiding this research was a modified health system performance framework [27] with modifications informed by Tanahashi Bottleneck analysis [28], current literature, and expert input from the country and international levels. Sub-objectives of the study included understanding the following phenomena related to building resilience in CBHS with respect to the drought: impact of the drought on HEWs and health system, community engagement and participation, service delivery, adaptability, and preparedness. Semi-structured guides with open-ended questions were developed and used for FDGs and KIIs. The guides were not validated or pilot tested. Guides were tailored to the participant’s knowledge or role in the health system.

Data were collected in the participants’ languages of preference (ie, English, Afsomali, Amharic, or Tigrinya) and facilitated by semi-structured interview guides. For non-English speaking participants, we employed simultaneous translation conducted by a UNICEF staff member or community-level health worker. This allowed for in-depth probing and feedback between the participants and the researcher. FGDs were approximately 1 hour in length while KIIs lasted between 30–45 minutes. All interviews were audio recorded with participants’ permission, transcribed verbatim, and translated into English. Translations were checked for accuracy by UNICEF team members but were not returned to participants for verification. There was no prior relationship between data collectors and participants. Data collection occurred in October 2016.

### Analysis

Data were analyzed using a deductive-inductive approach which began with first-level coding of verbatim English transcripts in ATLAS.ti software [29]. Open codes were defined by the sub-objectives and conceptual framework of the interview guide. We identified emerging themes using content analysis embedded in a grounded theory approach, chosen for its ability to identify the interconnectedness of the data and areas of conflict and contradiction. Thematic content analysis began by open-coding data on the first level to identify successes and challenges or facilitators and barriers related to three a priori domains: 1) HEWs and resilience during shocks (drought), 2) community engagement and participation, and 3) service delivery and HEWs. Emerging themes were synthesized based on community and non-community perspectives to examine the differences and similarities between these two perspectives. The co-authors discussed potential differences in the application of a priori and emerging themes to reach a consensus on classification. Data were triangulated using method triangulation [30] across sub-objectives to identify salient themes that were discussed in building resilience in community-based health systems.

Ethical approvals were obtained through the University of British Columbia Behavioural Research Ethics Board (certificate H1502651) and the Ethiopian Public Health Institute Scientific and Ethical Review Committee (certificate SERO-016-9-2016).

## RESULTS

### Participant Description

A total of 94 people participated in this study, including 71 from the community and 23 from the non-community level (**Table 1**). Non-community participants were included among District (Woreda) and Ministry of Health representatives, UNICEF country office team members, and other partners (bilateral, multi-lateral, and international NGOs). More than 40% (n = 10) of non-community participants were from the district (Woreda) level. The largest group of participants from the communities were from the women’s groups at 37% (n = 26). Of the 71 community participants, 21 (29.5%) were from Somali, Sitti Zone, 19 (26.8%) from Hawassa, SNNPR, and 31 (43.7%) were from Tigray, Eastern Zone (**Table 2**).

**Table 1:** Description of participants from Ethiopia (n = 94)

Community Participants (n = 71)	n	Community participants
Community leaders/elders	13	18%
Women development groups/women	26	37%
Health extension workers	19	27%
Health care workers	12	17%
**Non-community Participants (n = 23)**	**n**	**Non-community participants**
District (Woreda)	10	43%
Ministry of Health representatives	2	9%
UNICEF	6	26%
Partners (bilateral, multilateral, iNGOs)	5	22%
Total	94	

iNGO – international non-governmental organization

**Table 2:** Community participants by perspective and geographical distribution

Perspective	Participants	Method	Participants
**Somali, Sitti Zone (21 participants)**			
Community	Village leaders and elders	FGD	9
Community	Women’s group	FGD	8
Zonal	Mobile health team nurse	KII	1
Facility	Nurses at health post	FGD	3
**Southern Nations, Nationalities, and Peoples' Region (19 participants)**			
Community	HEWs – rural	FGD	6
Community	HEWs – urban	FGD	6
Community	Women’s development group	FGD	6
Zonal	Zonal health officer	KII	1
**Tigray, Eastern Zone (31 participants)**			
Community	Women’s development group	FGD	12
Community	HEW – rural	FGD	5
Facility	Primary health care unit staff	FGD	8
Community	HEWs	KII	2
Community	Village Leaders	FGD	4
**Total**			71

HEW – health extension worker, FGD – focus group discussion, KII – key informant interview

Participants identified several themes regarding building resilience in community-based health systems during the drought response in Ethiopia.

#### Organized community groups linked to the health system via the primary health care unit

First, participants felt community groups that were well organized, active, and engaged with the health system facilitated a successful and timely drought response. Participants discussed how community groups identified problems and solutions during the drought response, alerted decision-makers on the changing needs of the communities, conducted surveillance, mobilized and educated communities, and held the health system accountable to meet their needs. Participants felt that strong community groups allowed for quick information exchange with the health system and improved self-reliance, lessening communities’ dependence on government support during the drought response (Quotes 1 and 2, **Table 3**). The advantages of strong communication networks and feedback mechanisms between the community groups and the health system were widely discussed. Participants felt regular meetings improved accountability to communities and provided pathways for communities to communicate their needs, thereby ensuring a more effective response (Quote 3, **Table 3**). Many participants felt community-led initiatives were more successful because they were implemented with community buy-in and in coordination with health workers and facilities (Quote 4, **Table 3**).

**Table 3:** Quotes from participants by theme

Quote number	Perspective	Theme	Quote
**Organized community groups linked to the health system**			
1	District health officer	Quick information exchange and community self-reliance (Success)	*Currently, the community is well organized, networked, and information exchange is fast. Hence, any problem is traced at the ground level and reported to all actors in time, and the feedback and response comes down from above. Additionally, many of the problems are solved in-house, just by the community itself.*
2	Women’s health development army volunteer	Community self-reliance (Success)	*We contribute 5 Birr [0.22USD] per month per member in order to help our fellow community members to have access to health care, and food support for those poorest of the poor households who do not have capacity to pay, especially during the drought situations. We try to help each other with what we have at hand (grains, or money) without any expectation for external support from the Government. Contributed “by ourselves” and used “to ourselves” without becoming burden to the government. The women’s development group wanted to become self-reliant and self-supporting community.*
3	HEW, Urban	Regular Meetings between communities and health system (Success)	*We have public forum in every quarter the community is giving an idea, a suggestion to improve, to strengthen the health service so the community is contributing in suggesting [and] recommending and giving feedback on the service quality. This was very important.*
4	Women’s health development army volunteer	Community led initiatives with the support of facilities and health workers (Success)	*We perform our job in close support and coordination with the health staff at primary health care unit and the health extension workers. Just like “Hand and Glove” scenario. By in turn, the health center staff do not execute their job without the support of women’s development group deep in the community.*
5	Zonal health officer	Competing priorities of communities (Challenge)	*During the drought time the needs of the community have totally changed. Rather than vaccinating their children, they search for the food. We talk about prevention of Acute Watery Diarrhea. Simply, they think about food because their needs shifted [during] the drought. They need water; they need food items to advance themselves rather than participating [in] health programs.*
6	HEW	Competing priorities of communities (Challenge)	*They were having a problem of surviving but at this [drought] time when we go to the house—when we try to teach them they didn’t accept us; having a hunger…we don’t have food to eat but you are telling us to make sanitation, to have personal hygiene, so our priority is getting food we are not living in a good condition.*
**Effective community health workforce within strong health systems**			
7	International partner	Importance of HEW program (Success)	*The existence of the health extension worker program down to the community level contributed a lot to address the emergency situation in Ethiopia and to tackle and to respond [to the] emergency. What they have done during diarrhoea and drought—this is the most important lesson. They have a value to address such type of emergency as fast as possible*
8	UNICEF country office participant	Importance of HEW program identifying most marginalized and providing data (Success)	*The backbone of the drought responses was the nutrition concern. We can't find children without health extension workers. We can't treat children without them. We can't get data without them.*
9	HEW	Increased workload during drought (Challenge)	*During the drought, outbreaks occurred like Scabies and AWD [acute watery diarrhea/cholera]. Hence it created a lot of pressure on the routine activities and on all of the health actors.*
10	Ministry of Health manager	Importance of strong health system-health service coverage (Challenge)	*People can resist because children are already vaccinated, the bed nets are already distributed and some of the cases were treated. That really helps to reduce the effect of the drought. Whenever the coverage is high, it will make the system resilient whenever the drought hits in.*
11	Zonal health administrator	Importance of strong health system-supply chain (Challenge)	*The factor which can hinder [sustainability is] the supply chain system. If you [create] awareness for individuals when they come to the health center if there is no medicine and other medical equipment, they can’t come back to this health facility. Empowering the community and ensuring the supply chain system is very important to sustain and to create resilient health system.*
**Adaptable human resource structures and service delivery models**			
12	HEW, PHCU	Flexible human resources and coordination between facilities and communities (Success)	*The PHCU staff and HEWs arrange themselves in way that all the households in villages can be covered during drought situations. While HEWs work at households and community level, the HWs can support static services at health post level flexibility of human resource allocation. This makes the work of HEWs easier*
13	HEW (rural)	Flexible human resources and coordination between facilities and communities (Success)	*If there is drought, our time and energy is invested on the mitigation efforts of the outbreak. Hence the routine activities are compromised. These gaps have been solved by making close coordination with the Health Center and other sectors to share roles and tasks.*
14	HEW (rural)	Flexible human resources and coordination between facilities and communities (Success)	*Women’s development groups cannot replace my job [the work of HEWs], but they can mobilize the community so that I can give the services easily such as immunization and screening. Because I may not cover all the household in my village in short time. I can go to some 15 to 18 houses per day, but with the support of these women development groups, we can reach for more houses and access many mothers in need of health care.*
15	HEW, PHCU	Flexible human resources and coordination between facilities and communities (Success)	*The activities both at outreach and at the health facility are done through proper coordination—the remaining staff covers the work of the other staff who went out to the community or Health Post. For example, I am working at ART [antiretroviral therapy] unit hence somebody covers my role while I am out, and vice versa. Everyone is generalist to cover the work of others both at community and at health facility. This makes our work easy to deliver the needed services*
16	UNICEF country office participant	Flexible human resources and coordination between facilities and communities (Success)	*[Examining] resilience of health systems from the malnutrition side, the decentralization—the number, the network and the task shifting are major elements which allowed the health system to absorb the increased cases due to different crises*
**Training and preparedness**			
17	HEW	Preparedness reducing outbreaks (Success)	*Due to thorough preparedness, there was no AWD [acute watery diarrhea/cholera) outbreak in this village. Due to close follow-up and coordination with community and [the] health facility, including tracing of contacts, like those coming from any funeral ceremonies in other [villages], fearing that the diseased person might be suspected AWD case. Actions such as handwashing and health education have been implemented thoroughly*
18	HEW	Preparedness and application of plans (Success)	*We have applied specific actions like how to prevent, mitigate and take any follow-up actions after the drought. There was strong preparedness plan and coordination structure, and worked a lot on hygiene and sanitation interventions.*
19	HEW	Preparedness (Success)	*Due to the good preparation, the impact of the outbreak is less than expected. Both Scabies and AWD are controlled. Had it been not well prepared, the problem, coupled with drought and malnutrition would have brought serious negative impact on the health of the community*
**Strong government leadership with decentralized decision making**			
20	International implementing partner	Lack of adaptation due to long term vision (Challenge)	*In the face of this massive shock, it was still a traditional development system which is based on very little quick adaptation or quick reactions and much more on longer term processes. And I think all the systems failed. Not because they were weak; it's just that they were overstretched.*
21	UNICEF country office participant	Lack of international funding for health system (Challenge)	*The theory is stronger than the reality or the practice on the ground. Most of the time the partners will give you money for development and give you money for emergency response but there is little money for rehabilitation which will support resilience, preparedness and rehabilitation*

HEW – health extension worker, PHCU – primary health care unit, HW – health worker, AWD – acute watery diarrhoea

Participants also described challenges for community groups during the drought response, which included competing community priorities and resources, the need to improve accountability, and top-down programming. Many participants felt communities had shifting or competing priorities during the drought response (eg, the need for food or water compromising participation in prevention efforts) which resulted in limited uptake of educational interventions and participation in health programming (Quotes 5 and 6, **Table 3**). Many community women felt they would have more confidence in the health system and would be more likely to participate if they saw the results of their feedback when their messages were delivered to higher levels of the health system. Non-community participants also discussed challenges in meeting the communities’ needs after engagement and described how some programs were imposed on communities rather than being done in a participatory manner due to the acuteness of shocks. Participants described difficulties in mobilizing resources to meet the communities’ needs due to mismatched priorities across partners, especially during a shock such as a drought.

#### Effective community health workforce within strong health systems

The most widely discussed success of the drought response was the role of HEWs within strong health systems. Many participants described how HEWs’ roles prior to the drought were expanded beyond prevention, health promotion, and selective curative health services. These broadened responsibilities included providing drugs, immunisations, reducing newborn sepsis, and treating pneumonia, malaria, and diarrhoea, alongside their roles as community engagers and mobilizers. Examples of community mobilization included organizing and leading community groups, empowering communities, and training community development teams. Participants also described the importance of HEWs in coordinating international partners and donors, identifying those community members in greatest need during the drought response, and providing data in a timely way (Quotes 7 and 8, **Table 3**).

Participants identified diverse barriers and facilitators to HEWs performing their duties, which fell into broad themes related to populations and communities they served, HEW factors, and health system factors. Many participants felt HEWs experienced increased workloads during the drought and suggested that more HEWs should be hired and that HEWs needed to be better supplied, appropriately trained, given more incentives, and have improved mobility to cover larger distances. Many participants highlighted the importance of strong health systems within which HEWs work, including strong supply chains, committed facility-based health care workers, social support systems, and health service coverage (Quotes 9 and 10, **Table 3**). Many non-community participants cautioned on a health system’s overreliance on HEWs and highlighted the importance of strong health systems before, during, and after a shock.

#### Adaptable human resource structures and service delivery models

Third, adaptable human resource structures and service delivery models within communities and facilities were discussed as determinants of a successful drought response. Task shifting and teamwork between mobile health units, HEWs, community groups, and facility-based health care workers (HCWs) were discussed as facilitators of resilience. Specific examples included: HCWs working in villages to provide technical support to HEWs, HEWs sharing the workload within communities, HEWs having the flexibility of being based in either facilities or communities as priorities shifted, and HCWs being multiskilled to perform facility duties and outreach activities. Participants felt adaptable human resources improved the ability to respond to multiple shocks concurrently, prevented outbreaks, and increased the geographic reach of health education, promotion, and service delivery. Many participants attributed the health system’s ability to absorb increased workloads during the drought to workload redistribution between HEWs and HCWs. This was described as having allowed the health system to meet the increasing demands of communities, expanding networks into communities, and integrating different sectors with strong referral pathways (Quotes 11-15, **Table 3**). Mobile health units were discussed as an effective, alternative service delivery modality strategy to expand services and target the response to the populations who needed it most. This included pastoralists, those living in hard-to-reach areas, and internally displaced populations. Mobile health units were often discussed as a tool for filling a critical gap to meet the needs of migratory and pastoralist communities who would otherwise not have access to the health system. Lastly, regular meetings between HEWs and facility-based health workers allowed for opportunities to review the effectiveness of interventions and facilitated their ability to respond to the drought.

Challenges related to adaptable human resources included the suboptimal numbers of human resources during the drought and in the health system broadly, especially in remote regions. Participants also felt health centres needed to be well-equipped and resourced to meet the needs of communities, including better supplies, medication, and relief kits. Lastly, some participants also expressed concerns about the sustainability of financing mobile health units beyond the drought response, especially since many were donor-driven.

#### Training and preparedness

Fourthly, training and preparedness were widely discussed successes during the drought response. Participants felt that training communities, HEWs, and HCWs prior to and during the drought allowed them to respond appropriately and quickly to the needs of communities. This was reported as having lessened the drought’s impact on health outcomes, specifically on outbreaks of infectious diseases (Quotes 16-18, **Table 3**). Many non-community participants discussed the success of using evidence in planning the drought response. Participants described how lessons learned from previous droughts guided the advanced procurement of supplies. HCWs described prevention and mitigation strategies that prepared them for the drought which included drug acquisition and coordination done prior to the drought for reportable diseases (eg, scabies, water-borne diseases). Participants felt the early warning systems and health development plans allowed for evidence to be captured and disseminated quickly. Participants also discussed the successes of identifying the most marginalized villages and prioritizing them with constant evaluation of the gaps in services provided to them. Participants discussed the increased surveillance and strengthened reporting structures as successes that enabled the quick and effective deployment of supplies.

Although many participants discussed the advantages of preparing for anticipated outbreaks, unplanned ones were challenging (eg, dengue, chikungunya, and scabies). Participants felt that training communities and health workers on a wider array of possible threats, especially communicable diseases, would have facilitated a stronger response. Many participants felt there was a need for continuous risk assessment during an emergency to adequately prepare for any shock. Participants also highlighted the need for risk-informed programming, disaster reduction strategies, and policies that could be implemented during the drought response. Participants felt the finances and logistics needed to be in place from the lowest to the highest levels of the health system to adequately prepare for successful drought response.

#### Strong government leadership with decentralized decision making

Strong government leadership was a widely discussed success of the drought response. Many participants felt the government response was well-coordinated through multiple levels of the health system and across multiple sectors. Participants reported that the successes of the government’s response included the provision of financial resources, technical support, capacity building, and other resources to communities. Many participants discussed how the government guided partners to the neediest geographic areas and the advantage of the health sector being the command post where the response with partners was coordinated from higher to lower levels of the health system.

Decentralized decision-making during the drought response was also discussed as a success. Community elders and leaders described the success of communities coming together to decide how to allocate resources received from governmental and international donors. Decentralized decision-making to districts, facilities, and community levels was described to increase adaptability to meet the affected populations’ needs. Strong district-level support was also discussed as a facilitator of the response by mobilizing the communities.

Participants also identified challenges to the government response and decentralization. Many non-community participants felt that districts needed more decision-making power and resources for a more timely and effective response. Building district capacity for decision-making was also discussed as a method for fostering resilience. Some participants felt the government was focused on longer-term programming, which was a barrier to quick, adaptable health systems, but highlighted a need for both (Quote 19, **Table 3**). Many participants highlighted challenges related to differing priorities on the part of the government and international donors, which hindered the donors’ abilities to respond to the drought. Some felt the government not declaring an emergency was also a barrier to the international community mobilizing resources for the drought response. Participants also noted difficulties in raising money beyond the emergency response, leading to missed opportunities for rehabilitation, capacity building, and/or strengthening the health system (Quote 20, **Table 3**).

## DISCUSSION

Participants identified several important themes for building resilience in community-based health systems based on their experiences during the drought response in Ethiopia. Major themes included the importance of communities, decentralized health systems, training and multisectoral collaboration. The need for strong and adaptable health systems was highlighted across themes, focusing on features that would allow health systems to meet the needs of populations both in times of crisis and beyond. Community members highlighted the importance of feeling heard by decision-makers and the need for more health workers and resources, as well as ongoing training to enable communities to be prepared and respond quickly to future emergencies. Major priorities for non-community participants included a reduction in over-reliance on HEWs, stronger supply chains, and greater clarity in terms of priorities between governmental and non-governmental actors in crisis response.

Communities and HEWs were central to the success of the response across all themes and building their capacities must be prioritized to respond to various shocks to health systems. HEWs in this context have demonstrated key elements of resilience as defined by connecting the communities with “the people, relationships and local contexts that constitute health systems”, but must be further supported and strengthened [31]. This includes increased numbers of HEWs implemented alongside broader health system improvements (eg, investments in health posts, supply chains, information systems, supportive supervision, and strong referral networks) [32]. Additionally, as discussed elsewhere, strong self-reliant community groups linked to the health system can build the community’s adaptive capacity to respond to shocks such as cyclic droughts [2,33,34].

Previous research has described lessons learned at the community level during other health shocks (eg, Ebola virus disease), including the need to engage communities early and build trust [35,36]. In contexts where communities were consulted late in the response and in fragmented ways across diverse partners (eg, in Sierra Leone and Liberia during the Ebola virus outbreaks), community trust in the response was limited [36,37]. The Ethiopian health system was uniquely well-placed to engage in these early trust-building activities because community groups and health workforces were supported by the government and engaged relatively uniformly from the inception of the drought response. Despite this, our findings indicate that “top-down” programming, shifting and competing priorities during the drought, and lack of accountability were persistent challenges to building resilience within community health systems.

As we continue to unpack what is meant by building resilience in community-based health systems, there is an urgent need to better understand the intersection of communities and their health systems, as well as how communities’ roles can best facilitate resilience. As extreme weather patterns and outbreaks of infectious diseases continue to place increasing pressure on already strained health systems, community groups have a critical role to play in filling important resource and service gaps and in contributing to crisis responses. However, a lack of supervision and incentives for community groups, low perceptions of community inclusiveness, and shortages of community health workers have been linked to decreased service utilization in some regions, especially for MNCH services [8,38].

Well-resourced, supported, and trained health personnel have been identified as key in resilient health systems, with the World Health Organization stating “resilient health systems can only be achieved with a strong health workforce” [39]. Therefore, to build resilience in community-based health systems, we recommend investments in communities, especially the community health workforce and community groups.

Decentralized decision-making and training were often discussed as determinants of resilience. Communities where initiatives were led at the local-level (as opposed to top-down responses) were described as more empowering and effective in this context. With districts having the decision-making power to reallocate resources as needed while being supported by well-trained individuals and groups, communities could address a broad range of threats. Flexibilities in resource allocation would allow for rapid scale-up of acute drought responses in areas where they are most needed. Building resilience moved beyond the health sector, with intersectoral engagement also playing a key role in building resilience by addressing population needs such as food and water. The importance of this preparedness and flexibility in enabling an agile emergency response is particularly relevant considering the COVID-19 pandemic. Despite lessons from previous shocks, where preparedness was key for a successful response and continuation of essential services, the COVID-19 emergency response was woefully inadequate [40,41] and resulted in major disruptions to other areas of the health system, including the provision of services for HIV, malaria, and tuberculosis [40]. In a review of preparedness and response plans from 106 countries, considerations for subnational non-COVID essential health service delivery were only present in 34% of countries and less than half had included a mechanism that considered health system-wide services in their emergency planning [41]. As emergent health threats of pandemic potential continue to arise in the years ahead, planning and preparedness with communities across health systems must be prioritized, with a key focus on community health workforce and community groups.

Our study has several strengths and limitations. A major strength was that, to our knowledge, this is the first study to explore the drought response in building resilience in CBHS from diverse geographic regions and communities in Ethiopia. This allowed us to document relevant themes across communities and experiences. The diversity in participants across the health system fostered our understanding of the drought response from a cross-cutting health system perspective. We limited recall bias by interviewing participants during the drought crisis. However, there are some limitations to our study and the applicability of the findings. Our findings may not be generalizable to other regions of Ethiopia besides the three included in our study. Additionally, we may have captured themes related to the drought response at the specific time of our study. As the drought continued, the ongoing impact on workloads and resources may have resulted in different findings. Lastly, in areas with historical tension with the government, and given the political climate in Ethiopia, it is possible some respondents were not entirely forthcoming in their responses, which could have led to a reporting bias.

## CONCLUSIONS

Building resilience in community-based health systems during the drought in Ethiopia was facilitated by strong communities and community health workforces within health systems that were adaptable to the population’s needs. Further research is needed to understand the determinants of building resilience from various types of shocks in multiple contexts, especially focusing on harnessing the power of communities as reservoirs of resilience. There is an increasingly urgent need to move our understanding of resilience from theory to operationalization as our changing world will need to respond to crises.
